# Treatment with the calcineurin inhibitor and immunosuppressant cyclosporine A impairs sensorimotor gating in Dark Agouti rats

**DOI:** 10.1007/s00213-020-05751-1

**Published:** 2020-12-21

**Authors:** Jan Brosda, Thorsten Becker, Mathis Richter, Marie Jakobs, Tina Hörbelt, Ivo Bendix, Laura Lückemann, Manfred Schedlowski, Martin Hadamitzky

**Affiliations:** 1 Institute of Pharmacology and Toxicology, School of Veterinary Medicine, Freie Universität Berlin, 14195 Berlin, Germany; 2 Institute of Biology, Department of Neurophysiology, Freie Universität Berlin, 14195 Berlin, Germany; 3 Institute of Medical Psychology and Behavioral Immunobiology, University Hospital Essen, University of Duisburg-Essen, 45122 Essen, Germany; 4 Department of Pediatrics I/Experimental perinatal Neuroscience, University Hospital Essen, University of Duisburg-Essen, 45122 Essen, Germany; 5 Department of Clinical Neuroscience, Osher Center for Integrative Medicine, Karolinska Institutet, 17177 Stockholm, Sweden

**Keywords:** Cyclosporine A, Calcineurin, Prepulse inhibition, Sensorimotor gating, Drug side effects

## Abstract

**Rationale:**

Calcineurin is a protein regulating cytokine expression in T lymphocytes and calcineurin inhibitors such as cyclosporine A (CsA) are widely used for immunosuppressive therapy. It also plays a functional role in distinct neuronal processes in the central nervous system. Disturbed information processing as seen in neuropsychiatric disorders is reflected by deficient sensorimotor gating, assessed as prepulse inhibition (PPI) of the acoustic startle response (ASR).

**Objective:**

Patients who require treatment with immunosuppressive drugs frequently display neuropsychiatric alterations during treatment with calcineurin inhibitors. Importantly, knockout of calcineurin in the forebrain of mice is associated with cognitive impairments and symptoms of schizophrenia-like psychosis as seen after treatment with stimulants.

**Methods:**

The present study investigated in rats effects of systemic acute and subchronic administration of CsA on sensorimotor gating. Following a single injection with effective doses of CsA, adult healthy male Dark Agouti rats were tested for PPI. For subchronic treatment, rats were injected daily with the same doses of CsA for 1 week before PPI was assessed. Since calcineurin works as a modulator of the dopamine pathway, activity of the enzyme tyrosine hydroxylase was measured in the prefrontal cortex and striatum after accomplishment of the study.

**Results:**

Acute and subchronic treatment with the calcineurin inhibitor CsA disrupted PPI at a dose of 20 mg/kg. Concomitantly, following acute CsA treatment, tyrosine hydroxylase activity was reduced in the prefrontal cortex, which suggests that dopamine synthesis was downregulated, potentially reflecting a stimulatory impact of CsA on this neurotransmitter system.

**Conclusions:**

The results support experimental and clinical evidence linking impaired calcineurin signaling in the central nervous system to the pathophysiology of neuropsychiatric symptoms. Moreover, these findings suggest that therapy with calcineurin inhibitors may be a risk factor for developing neurobehavioral alterations as observed after the abuse of psychomotor stimulant drugs.

## Introduction

Disruptions in attention and cognition are seen in patients with idiopathic schizophrenia and during psychotic states induced by stimulants, such as methamphetamine (Druhan et al. 1998). Impairments in sensorimotor gating, the ability of an organism to filter irrelevant sensory information, are evident in these patients, as well (Geyer et al. 2001). Sensorimotor gating is measured as prepulse inhibition (PPI) of the acoustic startle response (ASR) and uses almost identical procedures in humans and rodents (Braff et al. 2001; Koch 1999; Swerdlow et al. 2001; Swerdlow et al. 2000). Schizophrenic patients show impairment in PPI and similar deficits can be produced in rats experimentally by a variety of drugs, e.g., dopamine receptor agonists such as methamphetamine (Geyer et al. 2001; Hadamitzky et al. 2011; Hutchison and Swift 1999). While experimentally induced PPI deficits are clearly not animal models of schizophrenia or stimulant-induced psychosis, they may serve as endophenotypes or physiological markers which are associated with the disease (Cadenhead et al. 2002; Swerdlow et al. 1994, 2000).

Calcineurin is a Ca^2+^/calmodulin-regulated serine/threonine protein phosphatase that is widely expressed in the central nervous system (CNS) and T lymphocytes (Hubbard and Klee 1989). The most specific and well-known potent inhibitors of calcineurin are the small-molecule drugs cyclosporine A (CsA) and tacrolimus (FK506). Due to their immunosuppressive action, these compounds are commonly used in clinical routine for the prevention of graft rejection after kidney, liver, and heart transplantation, as well as for treatment of inflammatory autoimmune diseases such as rheumatoid arthritis, or psoriasis (Halloran 2004; Lindenfeld et al. 2004; Taylor et al. 2001). Calcineurin inhibitors interact with cytoplasmic receptors such as cyclophilins and FK binding proteins (FKBPs) to form an immunophilin-immunosuppressant complex that binds calcineurin and inhibits its activity by sterically hindering the access of substrates to the catalytic site (Liu et al. 1992). Blockade of cellular calcineurin in T lymphocytes by both CsA and FK506 prevents dephosphorylation of the nuclear factor of activated T cells (NFAT), its transfer to the nucleus (Tedesco and Haragsim 2012), and its ability to initiate transcription of interleukin-2 (IL-2), interferon-gamma (IFN-γ), and related genes (Batiuk and Halloran 1997; Rusnak and Mertz 2000). In the CNS, calcineurin has been shown to play an important functional role in distinct processes like neurite extension, synaptic plasticity, or learning and memory (Winder and Sweatt 2001; Zeng et al. 2001).

Despite the known beneficial effects of calcineurin inhibitors and their widespread application in clinical conditions, little is known about unwanted neuropsychological side effects of this group of small-molecule immunosuppressants. Indeed, clinical evidence indicates that in patients treatment with calcineurin inhibitors may lead to an increased incidence rate of neuropsychiatric side effects such as disorientation, depression, aggression, paranoia, or psychosis (Chang et al. 2001; de Groen et al. 1987; Kahan 1994; Kahan et al. 1987; Lang et al. 2009; Lindenfeld et al. 2004). Moreover, it was demonstrated in rodents that acute administration of CsA decreased calcineurin activity in the brain and resulted in neurobehavioral activation, reflected as taste avoidance and elevated neuronal activity in the insular cortex and the amygdala (Pacheco-Lopez et al. 2013). Chronic treatment, however, increased levels of anxiety- and depressive-like behavior, and disturbed social behavior (Mineur et al. 2014; Sato et al. 2007). Importantly, several lines of evidence suggest that altered calcineurin signaling may contribute to the pathogenesis of schizophrenia or the development of schizophrenia-like symptoms (Takao and Miyakawa 2006). On one hand, calcineurin-mutant mice resembled a spectrum of forebrain-specific abnormalities remarkably similar to that observed in schizophrenic patients, such as increased locomotor activity, decreased social interaction, and impairments in prepulse inhibition, as well as working memory deficits (Miyakawa et al. 2003; Zeng et al. 2001). On the other hand, investigations identified a calcineurin-related gene (PPP3CC, which encodes for the calcineurin-γ subunit) as a potential schizophrenia susceptibility gene (Gerber et al. 2003). The finding that calcineurin is located downstream of the glutamate *N*-methyl-d-aspartate (NMDA) receptor signaling pathway and that it works as a modulator of the dopamine pathway (Lin et al. 2007; Takao and Miyakawa 2006) strongly suggests a possible association between calcineurin signaling and the traditional theories of schizophrenia pathophysiology: dopamine hyperfunction and/or glutamate hypofunction (Abi-Dargham and Moore 2016; Laruelle et al. 2003). This hypothesis is further supported by a case report of a schizophrenic patient, whose acute exacerbation of symptoms was strongly related to treatment with the calcineurin inhibitor FK506 (Lin et al. 2007). Evidence indicates that disturbances in frontal cortex dopamine may contribute to the occurrence of psychotic episodes (Carey et al. 1995), while psychostimulant-induced locomotion, one phenotype for psychotic symptoms in rodents, is associated with dopaminergic activation in the striatum (Kesby et al. 2018).

The present study investigated in healthy rats effects of acute and subchronic treatment with the immunosuppressant and calcineurin inhibitor CsA on sensorimotor gating. To gain insight on possible changes in central dopaminergic activity, the rate-limiting enzyme of dopamine synthesis, tyrosine hydroxylase (Daubner et al. 2011), was measured in the prefrontal cortex and the striatum.

## Methods

### Animals

Male Dark Agouti rats (200–230 g, correspondingly 50–60 days of age), a rat strain widely used in immunological research (Dimitrijevic et al. 2018), were obtained from Janvier (Le Genest-Saint-Isle, France) and group housed for an acclimation period of 1 week with ad libitum access to food and tap water. The vivarium was temperature (20 °C) and humidity (55 ± 5%) controlled. To allow the experiments to be conducted during the active phase of the rats’ awake/sleep cycle, it was maintained on a reversed 12-h dark, 12-h light cycle (7:00 a.m. to 7:00 p.m.). During the second week, animals were gently handled 5 min/day by the person performing the experiments. The animal facilities as well as the experimental procedures were in accordance with the National Institutes of Health and the Association for the Assessment and Accreditation of Laboratory Animal Care guidelines and were approved by the Institutional Animal Care and Use Committee (LANUV Düsseldorf, North Rhine-Westphalia).

### Drugs

The calcineurin inhibitor and immunosuppressant CsA (100 mg/mL; LC Laboratories, Woburn, USA) was dissolved in 100 μL ethanol (96%) and 900 μL Miglyol (Caelo, Germany). Prior to intraperitoneal (i.p.) administration, this stock solution was further diluted with sterile saline (0.9% NaCl, Braun, Germany) to adjust for the required drug doses of 5 and 20 mg/kg body weight with an injection volume of 1 mL. Rats of the control groups received an equivolumed, weight-adapted dose of the stock solution diluted with sterile saline. The administered doses are based on previous studies, verifying this drugs’ immunosuppressive properties and efficacy on progress and outcome in animal disease models (Hadamitzky et al. 2016; Lückemann et al. 2020).

### Prepulse inhibition (PPI) of the acoustic startle response (ASR)

Sensorimotor gating was assessed as PPI of the ASR using an automated SRLab startle system (San Diego Instruments, San Diego, CA, USA) as described elsewhere (Brosda et al. 2011; Hadamitzky et al. 2011). The sound-attenuated, ventilated chambers consisted of non-restrictive Plexiglas cylinders (9 cm in diameter) resting on a platform, mounted above a sensitive piezoelectric sensor. Vibrations of the cylinder caused by the whole-body ASR were transduced into analog signals and digitized and stored on a computer using SRLab software. At the beginning of each session, animals were placed into the chambers for a 5-min acclimatization period. White background noise of 70 dB lasted throughout the session. A test session consisted of 100 trials presented in a pseudorandom order with variable inter-trial intervals (7–23 s). The protocol consisted of 20 pulse-alone white noise trials with an intensity of 118 dB sound pressure level (startle stimulus; duration 40 ms), 10 prepulse trials (86 dB SPL, duration 20 ms), 10 NoStim trials, and 40 prepulse-pulse trials. The prepulse-pulse trials consisting of a 118-dB pulse preceded by a prepulse of 74, 78, 82, or 86 dB (20 ms duration) emitted 120 ms before the pulse onset. At the beginning (startle block 1) and end of the session (startle block 2), 10 pulse-alone trials were carried out to measure habituation to the startle stimulus. However, these trials were excluded from startle magnitude calculations. In general, data was measured during a 100-ms time window after stimulus onset and averaged for each animal and trial type. PPI of the ASR was calculated for prepulse-pulse trials as a percentage of pulse-alone startle magnitude ((mean startle magnitude for pulse-alone trials−mean startle magnitudes for prepulse-pulse trials)/mean startle magnitude for pulse-alone trials) × 100. Startle sessions were ~ 45 min in duration. After every test session, test chambers were cleaned with 70% ethanol to eliminate possible odor cues left by previous animals.

### Neurochemical measurement

**Fig. 1:**
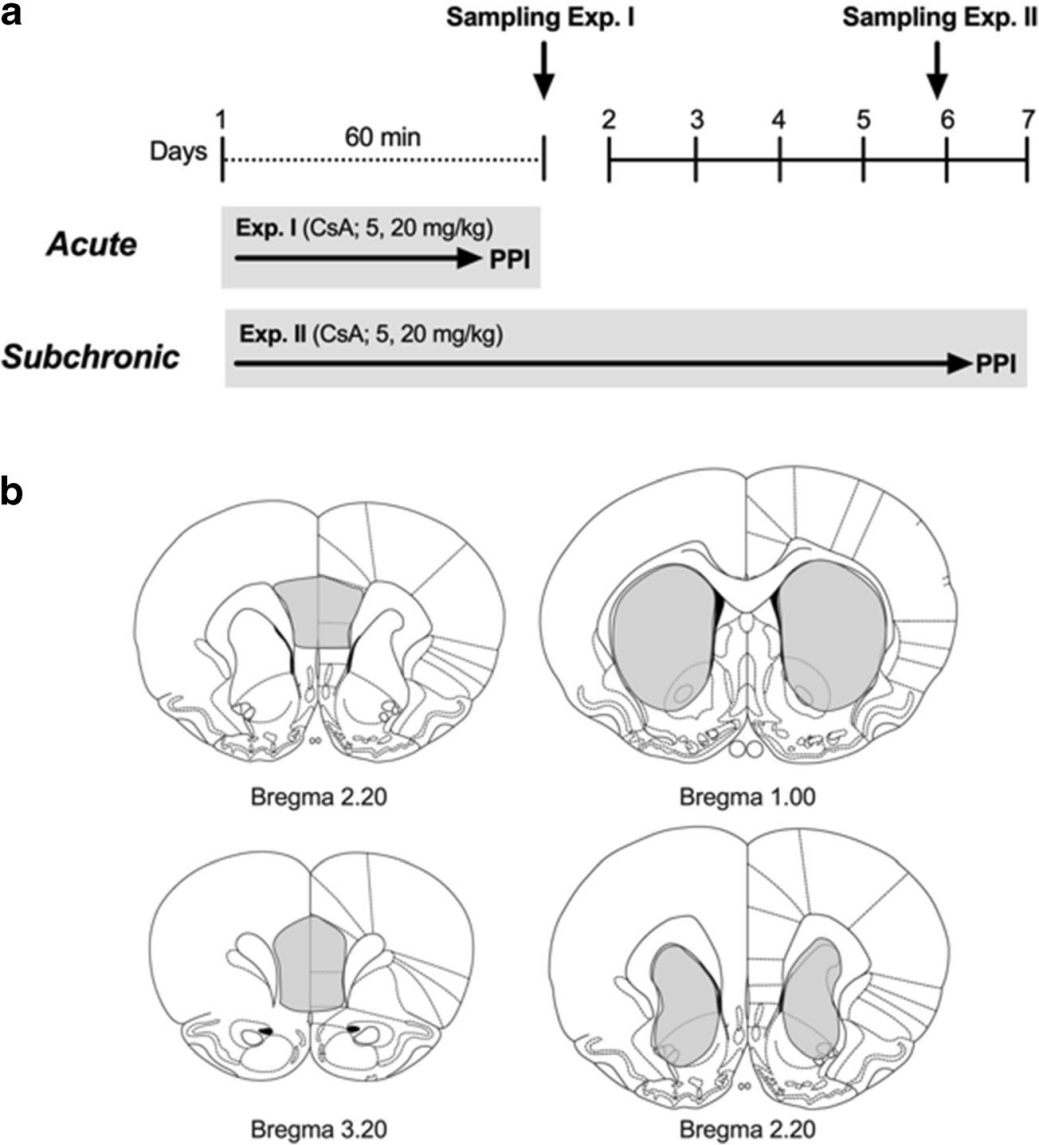
Experimental design (**a**). The study comprised two separate experiments. In the first experiment (group I), rats were tested for PPI of the ASR 60 min after an acute injection of cyclosporine A (CsA) at doses of 5 and 20 mg/kg. In the second experiment, distinct animals were treated with 5 or 20 mg/kg CsA for 7 days, once daily, before PPI of the ASR was assessed 60 min following the last injection. **b** Placement of punches. Immediately, after accomplishment of the last PPI session, respectively, animals were euthanized, and brains were quickly removed. As illustrated, coronal sections were cut on a cryostat, left and right prefrontal cortex (left hand series) and striatum (right hand series) were dissected from serial brain sections using a sample corer, and brain tissue was processed for protein expression analyses

For brain sample collection, anesthetized animals were euthanized immediately after assessment of PPI; brains were quickly removed, frozen on dry ice, and stored at − 80 °C. Using a freezing microtome (Microm HM560, Thermo Fisher Scientific, Walldorf, Germany), coronal brain sections of 200-μm thickness were cut at − 15 °C and placed on pre-chilled glass slides. Subsequently, the striatum as well as the prefrontal cortex (PFC; Fig. 1b) was dissected from serial brain sections using a micropunch technique (Cuello and Carson 1983). A pre-chilled stainless-steel sample puncher (internal diameter of 2 mm; Fine Science Tools, Heidelberg, Germany) was taken to obtain tissue samples of the left and right PFC and striatum. The optical tract, corpus callosum, and hippocampus served as anatomical landmarks to ensure comparable punch positions across animals (Paxinos and Watson 1998). Punches of each individual animal were pooled and proteins from the snap-frozen brain tissue were extracted utilizing freshly made radio-immuno-precipitation assay buffer (RIPA buffer; sc-24948, Santa Cruz Biotechnology) according to manufacturer’s protocol. Protein concentrations were calculated via BCA Protein Assay (Pierce Thermo scientific). For protein quantification, 30 μg protein per sample was diluted with 1× protein gel loading buffer (Roti®Load 1, CARL ROTH). Samples were boiled for 5 min at 95 °C, resolved on 8% SDS-PAGE gels, transferred to PVDF membranes, and probed with antibodies specific for TH (#58844), and phosphorylated (p)-TH (#2791) (both 1:1000, Cell Signaling). Antibody (#3700; 1:1000, Cell signaling) was used as loading control. Following membranes were washed and incubated with horseradish peroxidase–conjugated secondary anti-rabbit or anti-mouse antibodies ((#7074, #7076, both cell signaling). Proteins were visualized by chemiluminescent using Immobilon Western HRP Substrate detection reagents (Merck Millipore) on Fusion Imager (Vilber Lourmat). Protein signals were quantified by Image Lab™ Software (version 6.0.1, Bio Rad) and normalized for protein abundance of corresponding loading control β-actin. Sample sizes vary since non-evaluable probes had to be excluded.

### Experimental design

**Fig. 2:**
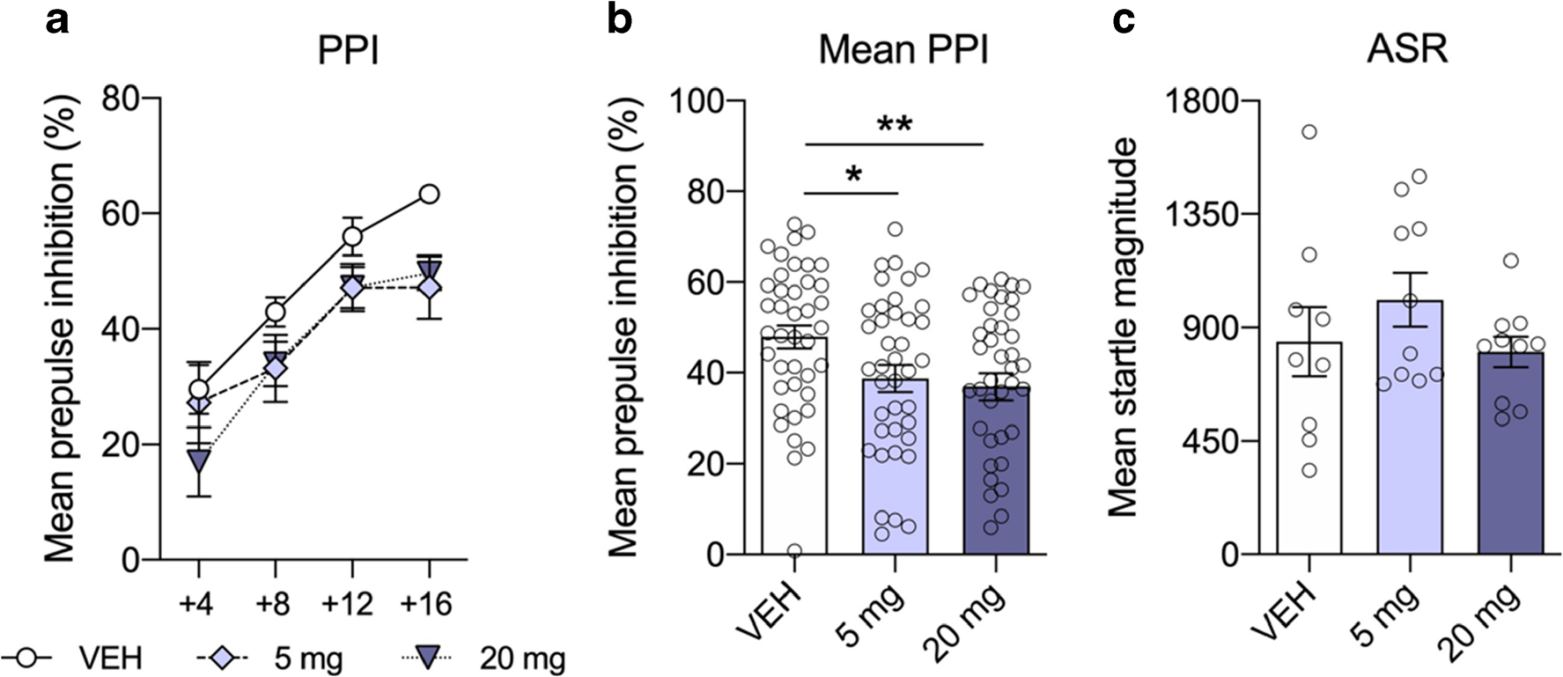
Acute treatment with the calcineurin inhibitor and immunosuppressant cyclosporine A on prepulse inhibition (PPI) of the acoustic startle response (ASR). The line plot shows percent PPI as a function of different prepulse intensities above background (**a**). Groups treated with CsA (5, 20 mg/kg) displayed diminished mean PPI scores compared to controls (**b**), while ASR did not differ between groups (**c**). Data are shown as scatter plot with mean ± SEM. Differences between groups are indicated by asterisk (ANOVA, Bonferroni post hoc; *p < 0.05; **p < 0.01; *n* = 8–10/group)

Central and peripheral activity and distribution of CsA 60 min following acute treatment, as well as after six consecutive daily i.p. injections of CsA, have already been confirmed (Hadamitzky et al. 2016; Pacheco-Lopez et al. 2013; von Horsten et al. 1998). Based on these findings, the present study comprised two separate experiments (Fig. 2). In the first experiment, effects of acute CsA (5 or 20 mg/kg) on sensorimotor gating were assessed. In a second experiment, the impact of subchronic treatment effects of CsA on sensorimotor gating (5 or 20 mg/kg CsA once daily for 7 days) before PPI was assessed 60 min following the last drug administration. Since previous works confirmed that group sizes of *n* = 7–10 are sufficient to gain statistical affects regarding central side effects of small-molecule immunosuppressive drugs (Hadamitzky et al. 2014, 2018; von Horsten et al. 1998), similar group sizes were used in the present approach. PPI testing was always performed under red-light illumination during the activity period of the animals. Thirty minutes prior to testing, animals were transferred to the experimental room to allow acclimation to the new surroundings. After each test session, the Plexiglas cylinders in the startle chambers were cleaned with 70% ethanol to eliminate possible odor cues left by previous animals. Immediately, after accomplishment of the last PPI session, respectively, animals were euthanized, and brains were removed, and processed for protein expression.

### Data analyses

The descriptive statistics are based on means, and variance is indicated by the standard error of the mean (SEM). Statistical analyses were conducted using SigmaPlot software (Version 12.3, SPSS, Chicago, IL, USA) and the level of significance was set at *p* < 0.05. Multiple dose experiments were analyzed using one-way analysis of variance (ANOVA) followed by Bonferroni corrections for pairwise comparisons.

## Results

### Prepulse inhibition of the acoustic startle response after acute treatment with CsA

ANOVA showed an effect for the factor *treatment* (*F*(2,81) = 3.545; *p* = 0.043) and for the factor *prepulse* (*F*(3,81) = 44.057; *p* < 0.001), but no *treatment* × *prepulse* interaction (*F*(6,81) = 0.989; *p* = 0.438), following acute injections with the immunosuppressant and the calcineurin inhibitor CsA (Fig. 2a). ANOVA also revealed a treatment effect on mean PPI calculated as average of all four prepulse conditions (*F*(2,117) = 5.395; *p* = 0.006). Post hoc testing revealed that mean PPI in animals treated with 5 mg/kg CsA (*p* = 0.033) and 20 mg/kg CsA (*p* = 0.009) was significantly reduced compared to vehicle controls (Fig. 2b). Acute CsA had no effect on mean ASR (ANOVA; [*F*(2,27) = 1.31; *p* = 0.286]; Fig. 2c).

### Tyrosine hydroxylase levels after acute treatment with CsA

**Fig. 3:**
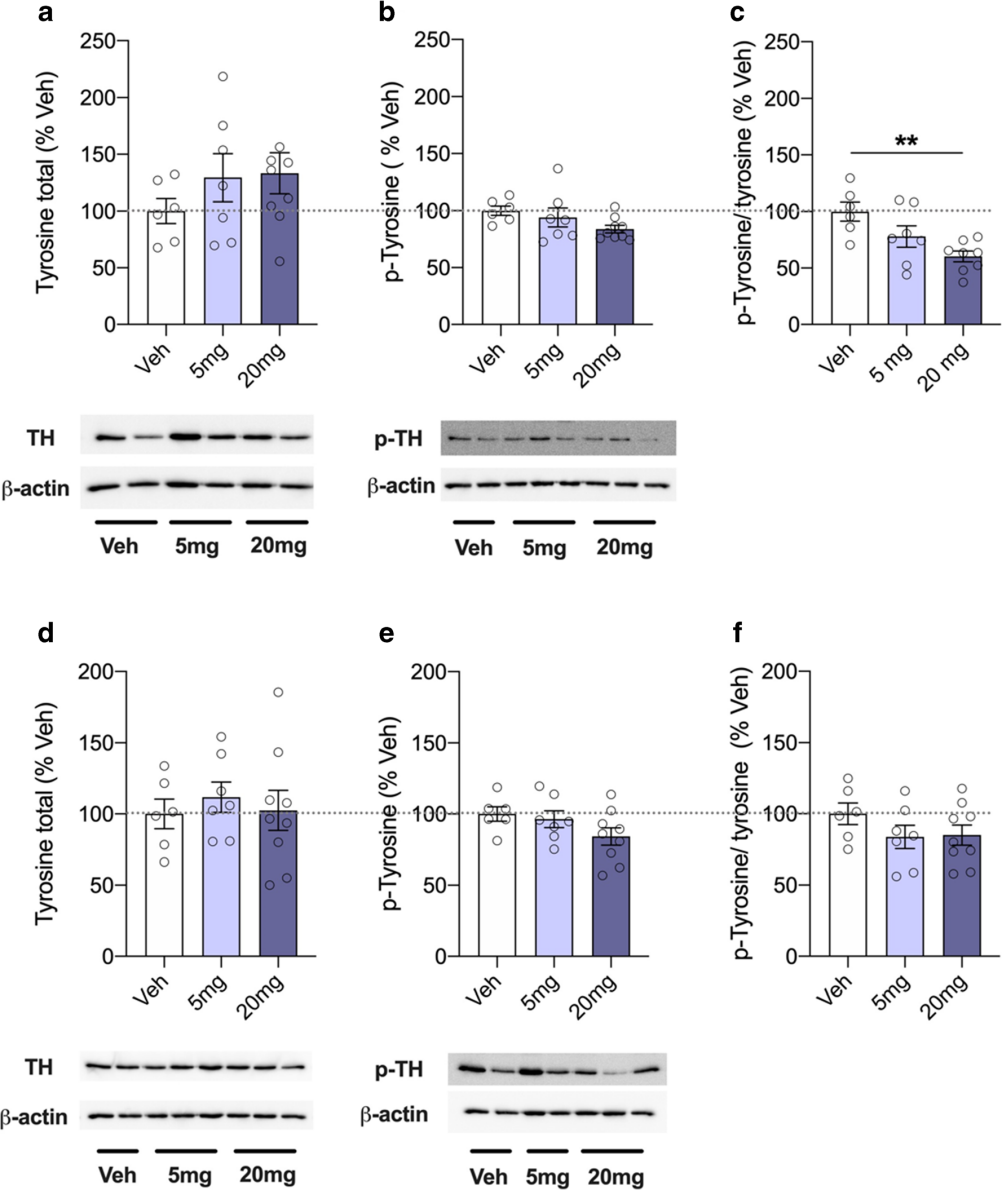
Western blot analysis of total tyrosine hydroxylase (TH) levels after acute treatment with the calcineurin inhibitor and immunosuppressant cyclosporine A (CsA; 5, 20 mg/kg). **a** Total TH, **b** phosphorylated (p)-TH, and **c** p-TH/TH ratio in the prefrontal cortex; **d** total TH, **e** p-TH, and **f** p-TH/TH ratio in the striatum of treated animals. Representative blot images show total TH, p-TH, and β-actin. Data are shown as scatter plot with mean ± SEM. Differences between groups are indicated by asterisk (ANOVA, Bonferroni post hoc; **p < 0.01; *n* = 6–9/group)

Expression of the rate-limiting enzyme in the synthesis of dopamine, evaluated as ratio between (p)-TH and total TH protein levels, was used as a marker of the dopaminergic activity (Struntz and Siegel 2018). While protein levels of total TH (*F*(2,89) = 0.906; *p* = 0.427; Fig. 3a) and p-TH (*F*(2,89) = 2.293; *p* = 0.128; Fig. 3b) did not differ between groups, ANOVA showed an effect of treatment in the p-TH/TH ratio (*F*(2,89) = 6.713; *p* = 0.0066; Fig. 3c). Post hoc analyses revealed a significantly reduced ratio in the 20 mg group compared to controls (*p* = 0.0036). In the striatum, however, neither protein levels of total TH (*F*(2,89) = 0.226; *p* = 0.799; Fig. 3d) and p-TH (*F*(2,89) = 2.056; *p* = 0.155; Fig. 3e) nor the p-TH/TH ratio (*F*(2,89) = 1.234; *p* = 0.312; Fig. 3f) differed between groups.

### Prepulse inhibition of the acoustic startle response after subchronic treatment with CsA

**Fig. 4:**
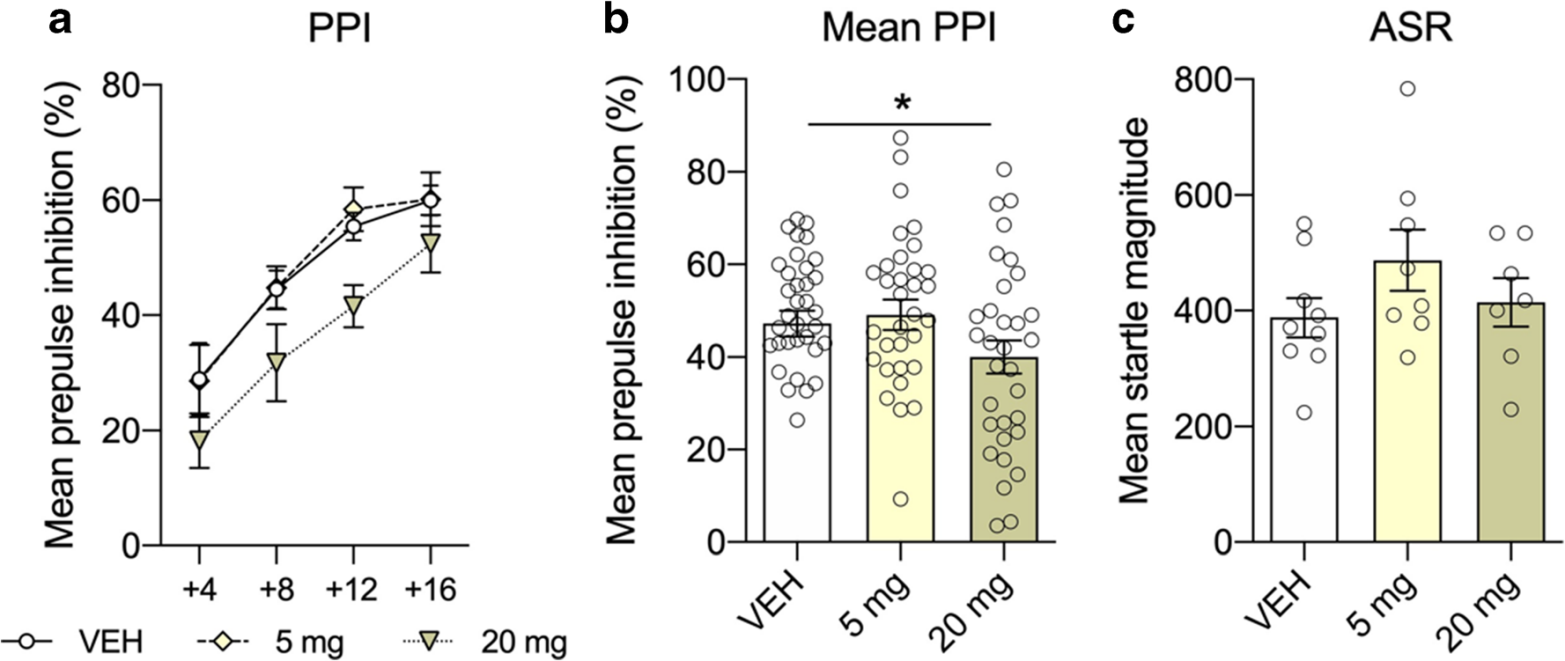
Subchronic treatment with the calcineurin inhibitor and immunosuppressant cyclosporine A (CsA) on prepulse inhibition (PPI) of the acoustic startle response (ASR). The line plot shows percent PPI as a function of different prepulse intensities above background (**a**). Daily injections with the calcineurin inhibitor for 7 days significantly diminished mean PPI in the 20 mg/kg group (**b**). At both doses, ASR was not affected (**c**). Data are shown as scatter plot with mean ± SEM. Differences between groups are indicated by asterisk (ANOVA, Bonferroni post hoc; *p < 0.05; *n* = 7–10/group)

In the subchronic experiment, following daily injection with CsA for 1 week, sensorimotor gating was impaired, as well (ANOVA; [*F*(2,89) = 3.705; *p* = 0.028]). Compared to controls, animals of the 20 mg/kg group displayed a reduced mean PPI (average of all four prepulse conditions; *p* = 0.022; Fig. 4b), whereas animals treated with the lower dose of 5 mg/kg did not differ from controls. As seen in the acute experiment, ASR was not affected (ANOVA; [*F*(2,117) = 0.935; *p* = 0.409]; Fig. 4c).

### Tyrosine hydroxylase levels after subchronic treatment with CsA

**Fig. 5:**
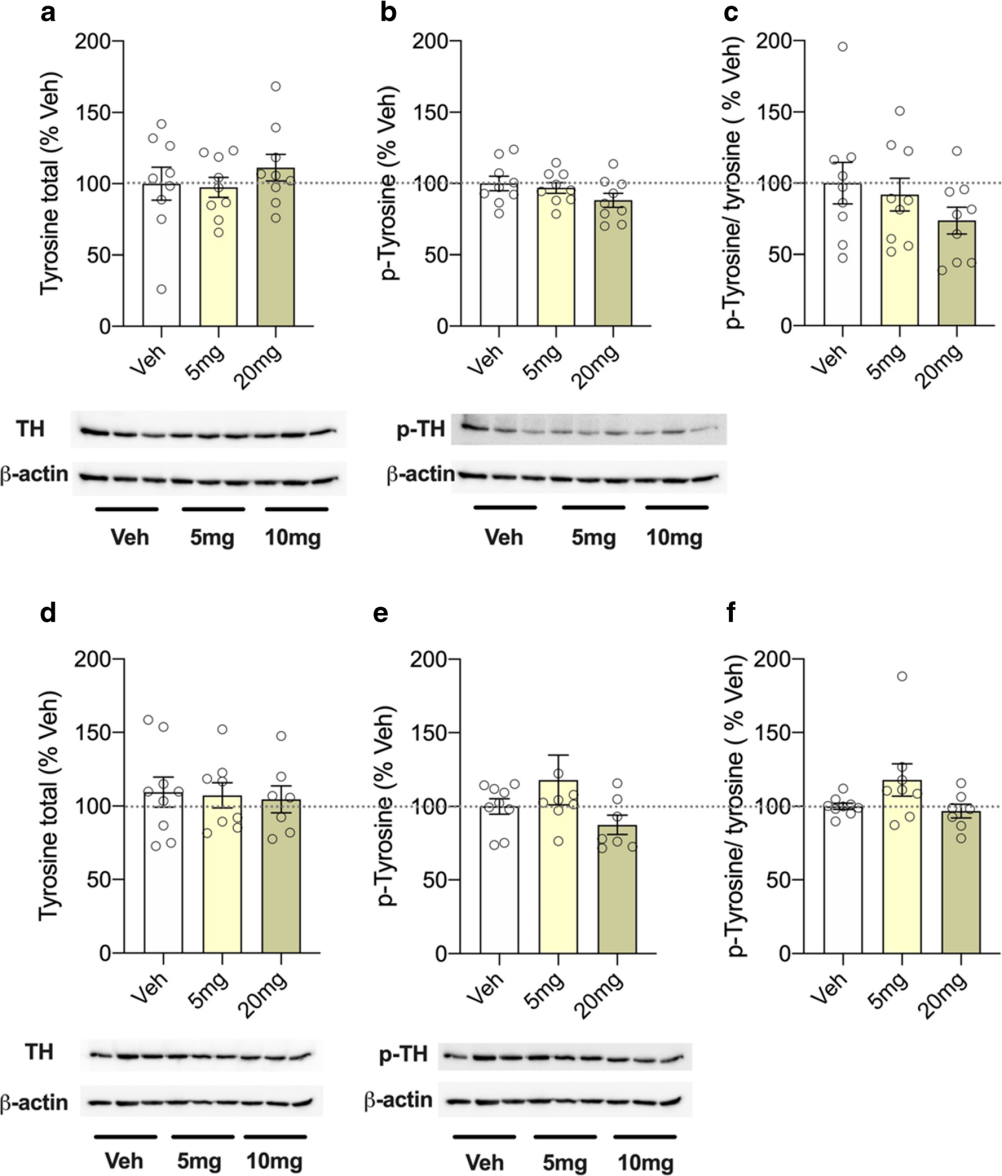
Western blot analysis of total tyrosine hydroxylase (TH) levels after subchronic treatment with the calcineurin inhibitor and immunosuppressant cyclosporine A (CsA; 5, 20 mg/kg). **a** Total TH, **b** phosphorylated (p)-TH, and **c** p-TH/TH ratio in the prefrontal cortex; **d** total TH, **e** p-TH, and **f** p-TH/TH ratio in the striatum of treated animals. Representative blot images show total TH, p-TH, and β-actin. Data are shown as scatter plot with mean ± SEM (ANOVA, Bonferroni post hoc; *n* = 8–10/group)

However, in the subchronic experiment, ANOVA showed no treatment effect on protein levels of total TH (*F*(2,89) = 0.617; *p* = 0.549; Fig. 5a) and p-TH (*F*(2,89) = 2.620; *p* = 0.094; Fig. 5b), or of the p-TH/TH ratio (*F*(2,89) = 1.541; *p* = 0.2432; Fig. 5c) in the prefrontal cortex. Likewise, ANOVA showed no group differences between protein levels of total TH (*F*(2,89) = 0.065; *p* = 0.9375; Fig. 5d), p-TH (*F*(2,89) = 1.9; *p* = 0.174; Fig. 5e), and p-TH/TH ratio (*F*(2,89) = 2.684; *p* = 0.0917; Fig. 5f) in the striatum.

## Discussion

This study found that acute and subchronic treatment with the calcineurin inhibitor and immunosuppressant CsA disrupted PPI at the dose of 20 mg/kg. We also observed reduced p-TH/TH ration in the prefrontal cortex following acute CsA treatment, potentially indicative for downregulated dopamine synthesis. These findings link impaired calcineurin signaling in the CNS to the pathophysiology of neuropsychiatric symptoms as observed after treatment with psychomotor stimulant drugs, characterizing therapy with calcineurin inhibitors as a risk factor for developing neurobehavioral alterations.

Patients with schizophrenia display impairments in measures of attention, cognition, and sensorimotor gating (PPI) (Geyer et al. 2001). Similar deficits can be induced experimentally in rodents using pharmacological manipulations (Braff et al. 2001; Koch 1999; Swerdlow et al. 2000, 2001). We show that acute and subchronic treatment with CsA resulted in impaired sensorimotor gating. This observation is consistent with findings, revealing diminished PPI in rats after acute and long-term treatment with amphetamines (Brunell and Spear 2006; Druhan et al. 1998; Fletcher et al. 2005; Hadamitzky et al. 2011; Mansbach et al. 1988; Tenn et al. 2003), or after direct infusion of dopamine into the mesolimbic dopamine system (Featherstone et al. 2007). These present findings are also congruent with outcomes seen in calcineurin-mutant mice. Among others, this forebrain-specific calcineurin knockout also led to impairments in PPI (Miyakawa et al. 2003; Zeng et al. 2001). Given that acute exacerbation of psychotic symptoms in a schizophrenic patient was strongly related to therapy with the calcineurin inhibitor FK506 (Lin et al. 2007), these data together suggest a link between altered calcineurin signaling in the CNS and the development of neuropsychiatric symptoms (Lin et al. 2007; Takao and Miyakawa 2006). However, effects after the different CsA treatment regimens were not consistent. While acute CsA disrupted PPI at both doses of 5 and 20 mg/kg (Fig. 2), subchronic treatment affected PPI only after daily administration of CsA at the dose of 20 mg/kg (Fig. 4). This may be attributed to the fact that 5 mg/kg CsA is considered as low therapeutic dose, exerting rather moderate inhibition on calcineurin. Apparently, these low dose effects are compensated during a subchronic treatment regimen and thus inefficient to block central calcineurin signaling leading to behavioral alterations. Studies revealed that deficits in PPI were abolished by administering the typical antipsychotic drug and dopamine D2 receptor antagonist haloperidol, pointing towards central dopamine hyperfunctioning as one cause for PPI disruption (Swerdlow et al. 1994; Swerdlow and Geyer 1993). Calcineurin is located downstream of the glutamate *N*-methyl-d-aspartate (NMDA) receptor signaling pathway (Lin et al. 2007; Takao and Miyakawa 2006) and it appears that calcineurin regulates both dopaminergic (Greengard 2001) and glutamatergic neurotransmission (Zeng et al. 2001). Phosphorylation of TH stimulates production of dopamine, but this enzyme is also rate limiting in the synthesis of this neurotransmitter (Daubner et al. 2011; Harada et al. 1996; Lindgren et al. 2000). Levels of TH and p-TH or rather the ratio of p-TH/TH may therefore be used as a marker for regional dopaminergic activity. We here show that impaired PPI following acute treatment with 20 mg/kg was also associated with decreased activity of TH in the prefrontal cortex (Fig. 3). These findings are consistent with studies, showing that psychomotor stimulants, which increased dopamine release from the ventral tegmental area and which in turn altered the striatal dopaminergic system (Sulzer 2011), decreased activity of TH in rats following acute drug exposure (Jedynak et al. 2002; Leonard 1977). Vice versa, central dopamine receptor blocker such as chlorpromazine and haloperidol increased activity of this enzyme (Leonard 1977). Moreover, it was shown that CsA administration mimicked cocaine’s actions and potentiated the locomotor responses to cocaine (Addy et al. 2008), suggesting that both compounds modulate their action at least partially via the same target structure, CaN.

However, surprisingly, we detected reduced TH activity neither in the striatum as seen after cocaine administration (Jedynak et al. 2002) nor in the striatum and the prefrontal cortex following subchronic treatment with CsA (Figs. 3 and 4). Regarding subchronic treatment, it may be possible that TH activity has potentially changed due to constant calcineurin blockade, since dopamine levels are rather quicker affected by CsA, as seen following acute treatment. At least, this was the case in a microdialysis study that evaluated effects of methamphetamine on extracellular levels of dopamine (Dobbs and Mark 2008). Given that the analyses of the striatum did not subdivide into the dorsal (caudate nucleus) and the ventral part (nucleus accumbens), potential effects on TH activity on either one of these structures may have been missed, in general. Nevertheless, we hypothesize that reduced TH activity following acute administration of CsA reflects (homeostatic) downregulation of catecholamine synthesis, as seen with psychostimulants such as cocaine. Conclusively, as also discussed as one traditional theory of schizophrenia pathophysiology (Abi-Dargham and Moore 2016; Laruelle et al. 2003), we assume that CsA-induced diminished calcineurin signaling increased cortical dopamine turnover, responsible with the observed PPI deficit (Carey et al. 1995).

### Conclusion and future directions

Patients suffering from autoimmune diseases or who underwent organ transplantation require regular, often life-long, immunosuppressive medication. It is assumed, and proven in some cases, that treatment with certain immunosuppressive drugs is frequently associated with the emergence of central side effects. The present study confirmed this hypothesis, showing that acute and subchronic treatment with the calcineurin inhibitor CsA led to deficient sensorimotor gating. Additionally, reduced TH activity was observed in the prefrontal cortex, indicating that central blockade of calcineurin by CsA possibly affected dopamine turnover.

However, a limitation of the study is that besides other markers for dopaminergic activity only activity of TH was measured. We initially intended to verify calcineurin blockade by blotting NFAT, but this approach failed due to limited detection profile. Despite the fact that following amphetamine administration increased extracellular dopamine concentrations were found in both striatal areas, the nucleus accumbens (ventral part) and the caudate nucleus (a dorsal part), these effects were more pronounced in the accumbens (Di Chiara and Imperato 1988). Similarly, injections of the dopamine antagonist haloperidol into the accumbens abolished amphetamine-induced locomotor activity, while injections into caudate had no effect (Pijnenburg et al. 1975). Against this background, a more detailed analysis of dorsal vs. ventral striatum or analyses of dopamine turnover and dopamine transporter expression (DAT) would have been advantageous. Microdialysis studies may therefore extend these initial findings by investigating the exact role of dopamine in distinct brain areas. Given that rodent models have demonstrated cognitive dysfunctions due to aberrant dopaminergic signaling in the medial prefrontal cortex (Castner et al. 2004), future studies may also address this issue by analyzing whether immunosuppressive compounds affect working memory in tests such as the radial maze, the Barnes maze, or the Morris water maze (Bendix et al. 2019).

Even though the exact mechanisms behind the observed neurobehavioral changes of CsA are still unclear, our findings support experimental and clinical evidence linking impaired calcineurin signaling in the CNS to alterations in dopamine activity and PPI deficits. Importantly, data regarding pharmacological options of treating neuropsychiatric side effects of, e.g., transplant patients are very sparse. It is known, however, that CaN-free immunosuppression with everolimus in heart transplant recipients significantly improvement CaN-induced side effects (Rothenburger et al. 2007). However, despite the availability of a wide range of small-molecule drugs with less side effects (Bosche et al. 2015), calcineurin inhibitors such as CsA are still first-choice drugs for the treatment of several immune-associated diseases. The present data indicate that therapy with calcineurin inhibitors may be classified as a risk factor for developing neuropsychiatric alterations, thereby addressing the importance for investigating exact neurobiological mechanisms of action of immunotherapeutic drugs, frequently used in daily clinical routine.

## References

[CR1] Abi-Dargham A, Moore H (2016) Prefrontal DA transmission at D1 receptors and the pathology of schizophrenia. Neuroscientist 9:404–416 10.1177/1073858403252674 14580124

[CR2] Addy NA, Bahi A, Taylor JR, Picciotto MR (2008). Administration of the calcineurin inhibitor cyclosporine modulates cocaine-induced locomotor activity in rats. Psychopharmacology.

[CR3] Batiuk TD, Halloran PF (1997) The downstream consequences of calcineurin inhibition. Transplant Proc 29:1239–1240 10.1016/s0041-1345(96)00481-2 9123289

[CR4] Bendix I, Hadamitzky M, Herz J, Felderhoff-Müser U (2019). Adverse neuropsychiatric development following perinatal brain injury: from a preclinical perspective. Pediatr Res.

[CR5] Bosche K, Weissenborn K, Christians U, Witzke O, Engler H, Schedlowski M, Hadamitzky M (2015). Neurobehavioral consequences of small molecule-drug immunosuppression. Neuropharmacology.

[CR6] Braff DL, Geyer MA, Light GA, Sprock J, Perry W, Cadenhead KS, Swerdlow NR (2001). Impact of prepulse characteristics on the detection of sensorimotor gating deficits in schizophrenia. Schizophr Res.

[CR7] Brosda J, Dietz F, Koch M (2011). Impairment of cognitive performance after reelin knockdown in the medial prefrontal cortex of pubertal or adult rats. Neurobiol Dis.

[CR8] Brunell SC, Spear LP (2006). Effects of acute ethanol or amphetamine administration on the acoustic startle response and prepulse inhibition in adolescent and adult rats. Psychopharmacology.

[CR9] Cadenhead KS, Light GA, Geyer MA, McDowell JE, Braff DL (2002). Neurobiological measures of schizotypal personality disorder: defining an inhibitory endophenotype?. Am J Psychiatry.

[CR10] Carey RJ, Pinheiro-Carrera M, Dai H, Tomaz C, Huston JP (1995). L-DOPA and psychosis: evidence for L-DOPA-induced increases in prefrontal cortex dopamine and in serum corticosterone. Biol Psychiatry.

[CR11] Castner SA, Goldman-Rakic PS, Williams GV (2004). Animal models of working memory: insights for targeting cognitive dysfunction in schizophrenia. Psychopharmacology.

[CR12] Chang SH, Lim CS, Low TS, Chong HT, Tan SY (2001). Cyclosporine-associated encephalopathy: a case report and literature review. Transplant Proc.

[CR13] Cuello AC, Carson S, Cuello AC (1983). Microdissection of fresh rat brain tissue slices. Brain microdissection techniques.

[CR14] Daubner SC, Le T, Wang S (2011). Tyrosine hydroxylase and regulation of dopamine synthesis. Arch Biochem Biophys.

[CR15] de Groen PC, Aksamit AJ, Rakela J, Forbes GS, Krom RA (1987). Central nervous system toxicity after liver transplantation. The role of cyclosporine and cholesterol. N Engl J Med.

[CR16] Di Chiara G, Imperato A (1988). Drugs abused by humans preferentially increase synaptic dopamine concentrations in the mesolimbic system of freely moving rats. Proc Natl Acad Sci U S A.

[CR17] Dimitrijević M, Arsenović-Ranin N, Bufan B, Nacka-Aleksić M, Macanović ML, Milovanović P, Đurić M, Sopta J, Leposavić G (2018) Collagen-induced arthritis in Dark Agouti rats as a model for study of immunological sexual dimorphisms in the human disease. Exp Mol Pathol 105:10–22 10.1016/j.yexmp.2018.05.007 29792851

[CR18] Dobbs LK, Mark GP (2008). Comparison of systemic and local methamphetamine treatment on acetylcholine and dopamine levels in the ventral tegmental area in the mouse. Neuroscience.

[CR19] Druhan JP, Geyer MA, Valentino RJ (1998). Lack of sensitization to the effects of d-amphetamine and apomorphine on sensorimotor gating in rats. Psychopharmacology.

[CR20] Featherstone RE, Kapur S, Fletcher PJ (2007). The amphetamine-induced sensitized state as a model of schizophrenia. Prog Neuro-Psychopharmacol Biol Psychiatry.

[CR21] Fletcher PJ, Tenn CC, Rizos Z, Lovic V, Kapur S (2005). Sensitization to amphetamine, but not PCP, impairs attentional set shifting: reversal by a D1 receptor agonist injected into the medial prefrontal cortex. Psychopharmacology.

[CR22] Gerber DJ, Hall D, Miyakawa T, Demars S, Gogos JA, Karayiorgou M, Tonegawa S (2003). Evidence for association of schizophrenia with genetic variation in the 8p21.3 gene, PPP3CC, encoding the calcineurin gamma subunit. Proc Natl Acad Sci U S A.

[CR23] Geyer MA, Krebs-Thomson K, Braff DL, Swerdlow NR (2001). Pharmacological studies of prepulse inhibition models of sensorimotor gating deficits in schizophrenia: a decade in review. Psychopharmacology.

[CR24] Greengard P (2001) The neurobiology of dopamine signaling. Biosci Rep 21:247–269 10.1023/a:1013205230142 11892993

[CR25] Hadamitzky M, Markou A, Kuczenski R (2011). Extended access to methamphetamine self-administration affects sensorimotor gating in rats. Behav Brain Res.

[CR26] Hadamitzky M, Herring A, Keyvani K, Doenlen R, Krugel U, Bosche K, Orlowski K, Engler H, Schedlowski M (2014). Acute systemic rapamycin induces neurobehavioral alterations in rats. Behav Brain Res.

[CR27] Hadamitzky M, Bosche K, Wirth T, Buck B, Beetz O, Christians U, Schniedewind B, Luckemann L, Gunturkun O, Engler H, Schedlowski M (2016). Memory-updating abrogates extinction of learned immunosuppression. Brain Behav Immun.

[CR28] Hadamitzky M, Herring A, Kirchhof J, Bendix I, Haight MJ, Keyvani K, Luckemann L, Unteroberdorster M, Schedlowski M (2018). Repeated systemic treatment with rapamycin affects behavior and amygdala protein expression in rats. Int J Neuropsychopharmacol.

[CR29] Halloran PF (2004) Immunosuppressive drugs for kidney transplantation. N Engl J Med 351:2715–2729 10.1056/NEJMra033540 15616206

[CR30] Harada WJ, Haycock JW, Goldstein M (1996). Regulation of L-DOPA biosynthesis by site-specific phosphorylation of tyrosine hydroxylase in AtT-20 cells expressing wild-type and serine 40-substituted enzyme. J Neurochem.

[CR31] Hubbard MJ, Klee CB (1989). Functional domain structure of calcineurin A: mapping by limited proteolysis. Biochemistry.

[CR32] Hutchison KE, Swift R (1999). Effect of d-amphetamine on prepulse inhibition of the startle reflex in humans. Psychopharmacology.

[CR33] Jedynak JP, Ali SF, Haycock JW, Hope BT (2002). Acute administration of cocaine regulates the phosphorylation of serine-19, -31 and -40 in tyrosine hydroxylase. J Neurochem.

[CR34] Kahan BD (1994). Role of cyclosporine: present and future. Transplant Proc.

[CR35] Kahan BD, Flechner SM, Lorber MI, Golden D, Conley S, Van Buren CT (1987). Complications of cyclosporine-prednisone immunosuppression in 402 renal allograft recipients exclusively followed at a single center for from one to five years. Transplantation.

[CR36] Kesby JP, Eyles DW, McGrath JJ, Scott JG (2018). Dopamine, psychosis and schizophrenia: the widening gap between basic and clinical neuroscience. Transl Psychiatry.

[CR37] Koch M (1999). The neurobiology of startle. Prog Neurobiol.

[CR38] Lang UE, Heger J, Willbring M, Domula M, Matschke K, Tugtekin SM (2009). Immunosuppression using the mammalian target of rapamycin (mTOR) inhibitor everolimus: pilot study shows significant cognitive and affective improvement. Transplant Proc.

[CR39] Laruelle M, Kegeles LS, Abi-Dargham A (2003) Glutamate, dopamine, and schizophrenia. Ann N Y Acad Sci 1003:138–158 10.1196/annals.1300.063 14684442

[CR40] Leonard BE (1977). Drug-induced changes in brain tyrosine hydroxylase activity in vivo. Neuropharmacology.

[CR41] Lin Y, Sun IW, Liu SI, Lohel W, Lin YC (2007). Tacrolimus ointment-induced relapse of schizophrenia: a case report. Int J Neuropsychopharmacol.

[CR42] Lindenfeld J, Miller GG, Shakar SF, Zolty R, Lowes BD, Wolfel EE, Mestroni L, Page RL, Kobashigawa J (2004). Drug therapy in the heart transplant recipient: part I: cardiac rejection and immunosuppressive drugs. Circulation.

[CR43] Lindgren N, Xu ZQ, Lindskog M, Herrera-Marschitz M, Goiny M, Haycock J, Goldstein M, Hökfelt T, Fisone G (2000). Regulation of tyrosine hydroxylase activity and phosphorylation at Ser(19) and Ser(40) via activation of glutamate NMDA receptors in rat striatum. J Neurochem.

[CR44] Liu J, Albers MW, Wandless TJ, Luan S, Alberg DG, Belshaw PJ, Cohen P, MacKintosh C, Klee CB, Schreiber SL (1992) Inhibition of T cell signaling by immunophilin-ligand complexes correlates with loss of calcineurin phosphatase activity. Biochemistry 31:3896–3901 10.1021/bi00131a002 1373650

[CR45] Lückemann L, Stangl H, Straub RH, Schedlowski M, Hadamitzky M (2020). Learned immunosuppressive placebo response attenuates disease progression in a rodent model of rheumatoid arthritis. Arthritis Rheumatol.

[CR46] Mansbach RS, Geyer MA, Braff DL (1988). Dopaminergic stimulation disrupts sensorimotor gating in the rat. Psychopharmacology.

[CR47] Mineur YS, Taylor SR, Picciotto MR (2014). Calcineurin downregulation in the amygdala is sufficient to induce anxiety-like and depression-like behaviors in C57BL/6J male mice. Biol Psychiatry.

[CR48] Miyakawa T, Leiter LM, Gerber DJ, Gainetdinov RR, Sotnikova TD, Zeng H, Caron MG, Tonegawa S (2003). Conditional calcineurin knockout mice exhibit multiple abnormal behaviors related to schizophrenia. Proc Natl Acad Sci U S A.

[CR49] Pacheco-Lopez G, Doenlen R, Krugel U, Arnold M, Wirth T, Riether C, Engler A, Niemi MB, Christians U, Engler H, Schedlowski M (2013). Neurobehavioural activation during peripheral immunosuppression. Int J Neuropsychopharmacol.

[CR50] Paxinos G, Watson S (1998). The rat brain in stereotaxic coordinates.

[CR51] Pijnenburg AJ, Honig WM, Van Rossum JM (1975). Inhibition of d-amphetamine-induced locomotor activity by injection of haloperidol into the nucleus accumbens of the rat. Psychopharmacologia.

[CR52] Rothenburger M, Teerling E, Bruch C, Lehmkuhl H, Suwelack B, Bara C, Wichter T, Hinder F, Schmid C, Stypmann J (2007). Calcineurin inhibitor-free immunosuppression using everolimus (Certican) in maintenance heart transplant recipients: 6 months’ follow-up. J Heart Lung Transplant.

[CR53] Rusnak F, Mertz P (2000) Calcineurin: form and function. Physiol Rev 80:1483–1521 10.1152/physrev.2000.80.4.1483 11015619

[CR54] Sato Y, Takayanagi Y, Onaka T, Kobayashi E (2007). Impact of cyclosporine upon emotional and social behavior in mice. Transplantation.

[CR55] Struntz KH, Siegel JA (2018). Effects of methamphetamine exposure on anxiety-like behavior in the open field test, corticosterone, and hippocampal tyrosine hydroxylase in adolescent and adult mice. Behav Brain Res.

[CR56] Sulzer D (2011). How addictive drugs disrupt presynaptic dopamine neurotransmission. Neuron.

[CR57] Swerdlow NR, Geyer MA (1993) Clozapine and haloperidol in an animal model of sensorimotor gating deficits in schizophrenia. Pharmacology Biochemistry and Behavior 44:741–744 10.1016/0091-3057(93)90193-w 8451276

[CR58] Swerdlow NR, Braff DL, Taaid N, Geyer MA (1994). Assessing the validity of an animal model of deficient sensorimotor gating in schizophrenic patients. Arch Gen Psychiatry.

[CR59] Swerdlow NR, Martinez ZA, Hanlon FM, Platten A, Farid M, Auerbach P, Braff DL, Geyer MA (2000). Toward understanding the biology of a complex phenotype: rat strain and substrain differences in the sensorimotor gating-disruptive effects of dopamine agonists. J Neurosci.

[CR60] Swerdlow NR, Geyer MA, Braff DL (2001). Neural circuit regulation of prepulse inhibition of startle in the rat: current knowledge and future challenges. Psychopharmacology.

[CR61] Taylor PC, Williams RO, Maini RN (2001). Immunotherapy for rheumatoid arthritis. Curr Opin Immunol.

[CR62] Takao K, Miyakawa T (2006). Investigating gene-to-behavior pathways in psychiatric disorders: the use of a comprehensive behavioral test battery on genetically engineered mice. Ann N Y Acad Sci.

[CR63] Tedesco D, Haragsim L (2012) Cyclosporine: a review. J Transplant 2012: 230386. 10.1155/2012/230386 PMC3259474 22263104

[CR64] Tenn CC, Fletcher PJ, Kapur S (2003). Amphetamine-sensitized animals show a sensorimotor gating and neurochemical abnormality similar to that of schizophrenia. Schizophr Res.

[CR65] von Horsten S, Exton MS, Voge J, Schult M, Nagel E, Schmidt RE, Westermann J, Schedlowski M (1998). Cyclosporine A affects open field behavior in DA rats. Pharmacol Biochem Behav.

[CR66] Winder DG, Sweatt JD (2001). Roles of serine/threonine phosphatases in hippocampel synaptic plasticity. Nat Rev Neurosci.

[CR67] Zeng H, Chattarji S, Barbarosie M, Rondi-Reig L, Philpot BD, Miyakawa T, Bear MF, Tonegawa S (2001). Forebrain-specific calcineurin knockout selectively impairs bidirectional synaptic plasticity and working/episodic-like memory. Cell.

